# Raman Analysis of Tear Fluid Alteration Following Contact Lense Use

**DOI:** 10.3390/s19153392

**Published:** 2019-08-02

**Authors:** Angela Capaccio, Antonio Sasso, Giulia Rusciano

**Affiliations:** 1 Department of Physics E. Pancini, University of Naples Federico II, Complesso Univesitario Monte S. Angelo, Via Cintia, I-80126 Naples, Italy; 2 National Institute of Optics (INO)-National Research Council (CNR), Via Campi Flegrei 34, I-80078 Pozzuoli, NA, Italy

**Keywords:** tear fluid, Raman Spectroscopy, contact lens wearing

## Abstract

Tear fluid is a heterogeneous solution containing mainly proteins, lipids, mucins and electrolytes, which regulates the physiology of the human eye. The complex composition of tears can be altered in the presence of eye inflammations. The use of contact lenses is one of the most frequent causes of inflammatory responses of the eye, with the related discomfort often causing the wearer to give up using them. In this paper, we exploit the potentiality of Raman Spectroscopy to analyse the biochemical changes in tear fluid in a contact lens wearer. In particular, we analysed the tear fluid collected from a volunteer as a function of the wearing time for two types of monthly contact lenses (Hydrogel and Si-Hydrogel). Our experimental results show an alteration of the relative concentrations of proteins and lipids in both of the analysed cases. More importantly, our results highlight the diagnostic sensitivity of Raman analysis to select the proper contact lens type for each wearer and optimise the lens wearing conditions.

## 1. Introduction

Human tear fluid is a complex blend of both organic and inorganic components, synergically involved in carrying out many important functions for the eye, including lubrication, the cleaning of waste and particulate matter and protection against infective agents [1]. Changes in the biochemical composition of tears can lead to ocular discomfort and eye inflammation, which, in turn, give rise to further alterations in tear composition [2]. This clearly explains the increasing attention being paid to the analysis of this bodily fluid [3].

On the ocular surface, the tear film is arranged in three layers [4]. The lower layer is essentially a mucus layer, promoting the spread and stability of the second overlying aqueous layer, which contains protective agents (mainly proteins and antibodies) which guard against ocular infections. Finally, the uppermost layer is mainly composed of lipids secreted by the meibomian glands, which stabilise the tear film and reduce the tear evaporation rate.

This layered structure can be significantly altered by the wearing of contact lenses (CLs). The effect of CLs on the tear film is essentially twofold [5,6]. Firstly, absorption on the lens surface can reduce the levels of specific components in the tear film. Secondly, the contact of the lens with the ocular surface can stimulate the increase of specific film components. For instance, it has been reported that lower molecular weight proteins can be absorbed into the lens matrix, and in turn, the lens itself can stimulate cascade processes leading to the generation of additional proteins and peptides [5]. Clearly, the eyes response to CLs is quite complex, being obviously dependent on both the single wearers characteristics and from the properties of the lens [7,8]. In particular, lens–tear interactions are reported to be dependent on the lens material’s ionicity and water content. Maissa et al. [9] reported that while protein interaction is highly dependent on the ionicity of the material, this parameter does not influence the lens–lipid interaction, for which the material hydrophobicity is the main factor. Up to now, lens–tear interaction has been mainly investigated by analysing tear component deposition on CLs, while only a few studies report on the effect of CLs use on tears. Most of the investigations on this topic were performed by fluoro-photometry assays, photon correlation spectroscopy [10] or tear-film break-up time analysis [11,12]. Raman Spectroscopy (RS) is also emerging as a reliable tool for the analysis of tear composition. On the basis of the inelastic scattering of photons, this technique presents several advantages, including the reduced sample volume required, the lack of sample pre-treatment and the reduced interference from water, which has made RS quite popular for the analysis of body fluids.

RS has been previously used to analyse tears, typically spotted on a hydrophobic substrate and allowed to evaporate. This procedure, usually referred as drop coating deposition Raman spectroscopy (DCDRS), leads to concentrations of macromolecule samples, with consequent amplification of the detected Raman signal. As it is well known, tear fluid evaporation forms characteristic ferning patterns. Filik et al. [13,14,15] have analysed the distribution of macromolecules in this pattern, finding that proteins, urea, bicarbonate and lipids can be detected in the tear samples and that the distribution of these components in the drying pattern was dependent on their relative solubilises. More recently, tear fluid has been also analysed by Surface Enhanced Raman Scattering (SERS), providing insight into the presence of selected proteins such as lysozyme [16] and lactoferrin [17]. Moreover Choi et al. [18] performed a SERS characterisation of dried tears, in order to point out the spectral changes occurring in adenoviral conjunctivitis-infected subjects.

In this work, we analysed the effect of CLs use on the composition of tear fluid. The investigation was performed using DCDRS analysis of tear samples as a function of the wearing time of monthly CLs. Since changes in tear fluid biochemistry are reported to be subject dependent [5], it was necessary to analyse tear samples collected from the same donor. The effect of CLs use was investigated for two types of CLs materials: Hydrogel CLs (H-CLs) and Silicone Hydrogel CLs (SH-CLs). SH-CLs have become very popular in recent years because of their high oxygen permeability and hydrophobicity, and the reduced protein deposits on their surface [19].

Our experimental results reveal a reduction in the concentration of proteins with respect to lipids in the first five days of CLs use, followed by a substantial recovery of the initial tear fluid content after two weeks. This behaviour was found for both types of CLs materials. More importantly, our work demonstrates that RS can be fruitfully used to probe the complex biochemistry involved in the interaction between CLs and eyes.

## 2. Materials and Methods

### 2.1. Sample Collection

As previously mentioned, a single healthy volunteer was recruited for tear fluid collection. Informed consent was obtained from him, after a full explanation of the tear fluid collection procedure and the scope of the investigation. The volunteer declared that he was not a contact lens wearer, and was free from ocular infection. Tears (≈5 μL) were collected using a smooth edge sterile plastic transfer pipette, which was put in contact with the tear meniscus near the interior lacrimal channel. The collected sample was immediately dropped on a quartz slide and allowed to dry at room temperature for ≈10 min before Raman analysis.

### 2.2. Contact Lenses

In this study, we investigated two monthly contact lenses, both provided from Soleko S.p.A. In Table 1, the main characteristics of the CLs used in this work are listed.

### 2.3. Experimental Set-Up

Raman measurements were carried out using a confocal Raman microscope (WiTec Alpha 300), provided with a Nd-YAG laser at 532 nm as Raman excitation source. The laser beam was focused on the sample through an oil-immersion 100× objective (NA = 1.4). Back-scattered light was collected using the same objective, filtered by an edge filter and sent to the spectrometer through a 50 μm core fibre, also acting as a pinhole for confocal detection. The Raman radiation was detected using a thermoelectrically cooled charge-coupled device at the spectrometer exit. Raman spectra were acquired in the 300–4000 cm${}^{-1}$ spectral range with a resolution of ≈3 cm${}^{-1}$.

### 2.4. Raman Spectra Acquisition and Processing

In this work, we acquired Raman spectra in selected points and/or areas of the ferning region. Single spectra were acquired with 20 s of integration time while Raman spectral maps were performed on small areas (10 × 10 μm${}^{2}$) by recording 30 points for line and 30 lines for scan with a total of 900 spectra and using an integration time of 10 s per spectrum. Data pre-processing (smoothing, background subtraction, cosmic rays removal) was performed, in both cases, using Project 2.4 WiTec software.

## 3. Results and Discussion

### 3.1. Raman Analysis of Human Tears

**Figure 1:**
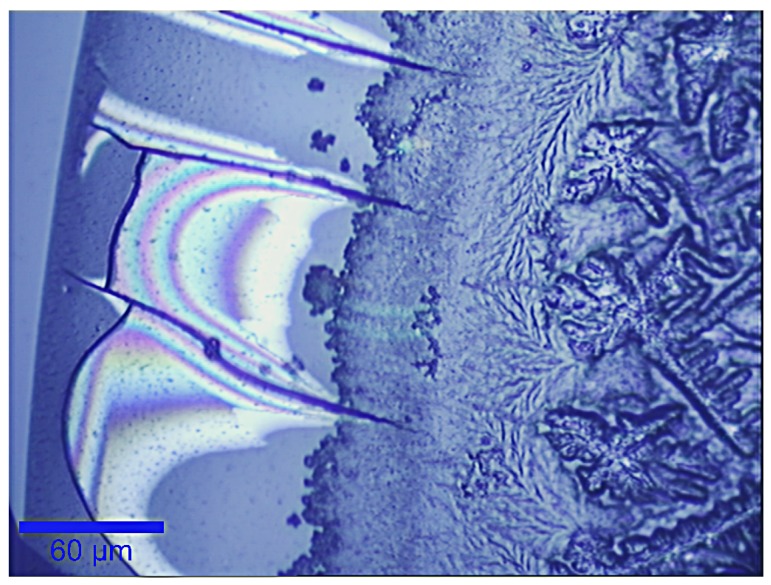
Bright-field image of a detail (edge) of a drying droplet of tear sample examined in this study. The scale bar is 60 μm.

In Figure 1, we show a bright-field image of the typical drying pattern assumed by one of the tear samples herein analysed, after the deposition on a quartz substrate.

**Figure 2:**
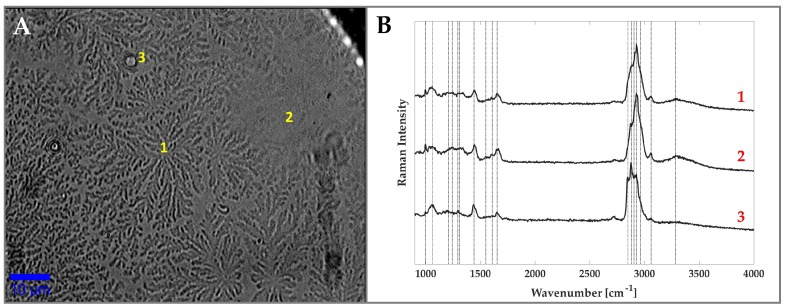
(**A**) Bright-field image of a tear deposit region and (**B**) Raman spectra corresponding to the points indicated in panel (**A**). The scale bar in the picture is 10 μm.

In this figure, the formation of a coffee-ring pattern along the edge and a crystallisation arrangement, described as “tear fern”, in inner part of the droplet are well visible; these have already been observed by many authors [20,21]. In particular, using RS Filik et al. [14] revealed the presence of proteins both in the outer ring (as also suggested by Pearce et al. [22]) and in the fern pattern. Moreover, the fern pattern also contained NaCl and urea in the inner part, and sodium bicarbonate at the edge. To the contrary, lipids, which represent the insoluble components of tears, were found to be spread out as debris throughout the whole drop, including the fern crystallisation region. This heterogeneity was also confirmed by our analysis. Figure 2 shows three Raman spectra acquired in different points in the middle of the tear fluid deposit, where the crystallisation is dominant. The position labelled “1” corresponds to the central part of a fern, position “2” corresponds to a point exhibiting a reduced fern crystallisation and position “3” corresponds to a microstructure of spherical shape with a diameter of about 2 μm (Figure 2A). The corresponding spectra were plotted in cascade in Figure 2B. A tentative assignment of the relevant signals is given in Table 2 [23,24,25]. Clearly, the different composition in the selected points is mirrored by the difference in the relative intensities of some peaks appearing in the spectra. In particular, the first and second spectra show prominent peaks related to proteins, with some contributions from lipids. Recent works reported on the presence of three main proteins in tears: lactoferrin, albumin and lysozyme [14,16]. According to our result, we observed the peaks at 1001, 1206, 1242, 1440, 1553, 1605 and 1665 cm${}^{-1}$, assigned to phenylalanine and tryptophan, which are characteristic of these protein spectra. Moreover, the spectra concerning points “1” and “2”, exhibit a broad band around 3300 cm${}^{-1}$, which can be assigned to OH stretch vibrations and it is reasonable to assume that these are due to residual water linked to soluble macromolecules (proteins, urea, salts).

The spectrum corresponding to position “3” shows an evident change in the relative intensities of the bands present in the region around ≈2800 cm${}^{-1}$, which are mainly associated with CH stretching in proteins and lipids. In particular, the peaks at 2845 and 2875 cm${}^{-1}$ are assigned to the CH${}_{2}$ stretching of lipids, whereas the peaks at 2930 and 2960 cm${}^{-1}$ are assigned to the CH stretch of lipids and proteins [23]. In view of this, we speculate that the observed increase in signals in the 2830–2900 cm${}^{-1}$ region could highlight the presence of a higher lipids concentration. Interestingly, the spectral features related to fatty acids also identified in the bands at 1064, 1296, 1301 and 1440 cm${}^{-1}$ show the same increase. This definitively allows us to identify the microsphere in position “3” as a lipid debris.

### 3.2. The Effect of Wearing CLs

As anticipated previously, the main goal of this paper is to check the possibility to monitor the biochemical changes in tear fluid induced by CLs use. To this purpose, a volunteer was engaged to wear both types of CLs described in the previous section. The experiment lasted two months in which the same volunteer wore the *LAC monthly wet* for the first month and the *AirHydra* during the second. During this time, tear fluid was periodically collected and spotted on a quartz coverslip for Raman analysis. In order to avoid any “memory effect” and to obtain a “blank sample” for the analysis, the volunteer did not wear any CLs for two weeks before the wearing of the CLs for the investigations.As explained above, sample analysis was performed by Raman imaging of the ferning region. In particular, for each sample, we acquired four Raman maps in the central part of the tear deposit, resulting in a total of 3600 spectra.

**Figure 3:**
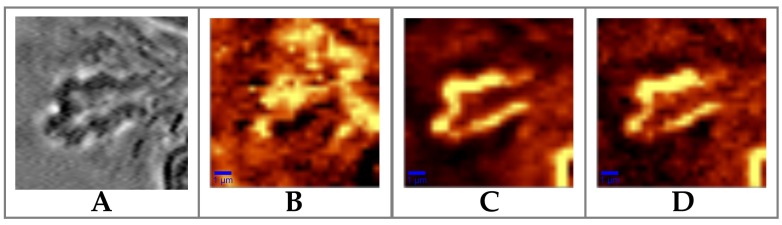
(**A**) Bright-field image of a ferning dentrite and relative Raman images obtained selecting the lipids band area (**B**) 2800–2900 cm${}^{-1}$, (**C**) the proteins band 2900–3000 cm${}^{-1}$ and (**D**) water band from 3100 to 3700 cm${}^{-1}$. The scale bar is 1 μm.

In Figure 3, we show the bright-field image of a ferning dentrite (part A) and the Raman scan images created by mapping three significant spectral regions: the 2800–2900 cm${}^{-1}$ region, which is mainly caused by CH${}_{2}$ bonds in lipids; the 2900–3000 cm${}^{-1}$ region, mainly caused by CH${}_{3}$ bonds in proteins; and the 3100–3700 cm${}^{-1}$ region, related to OH stretch (part B, C and D, respectively).

Interestingly, Figure 3C,D reproduce the border of the fern, revealing a major proteins and water soluble components contribution at the peripheral area compared to the lipids one, which is instead concentrated in the inner part (Figure 3B).

**Figure 4:**
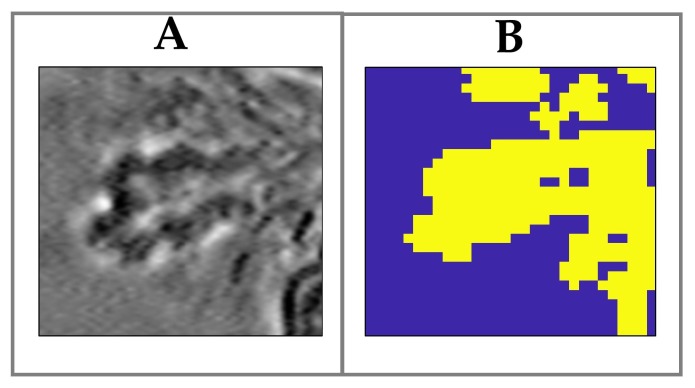
(**A**) Bright-field image of a ferning dentrite. (**B**) Mask used to locate the spectra corresponding only to the fern pattern (yellow region).

Spatial segregation of the different biochemical components in the tear drop is a crucial issue in order to gain information on the proteins/lipids components of the tear fluid. This feature is even more important when Raman data are used to compare the composition of different samples. As a matter of fact, a “bare average” of all the spectra acquired in a scan could be clearly affected by the filling fraction of the fern in the scanned area. More reliable outcomes can be instead obtained by introducing a rationale for selection of points in the Raman map to be used for the evaluation of sample composition. For this purpose, we developed an automatic routine to pick-up, from each Raman scan, only spectra acquired in points corresponding to the fern pattern. The selection of these points in the scan image was carried out using a custom-made Matlab code, based on Principal Component analysis (PCA) [26,27] applied to the acquired Raman images. In all the analysed Raman maps, the PC1 score map clearly distinguished the fern pattern from the surrounding background, according to the positive/negative sign of the scores. A similar result has been also reported by Filik et al. [13,14]. Therefore, a logical matrix was created to generate a mask which locates only spectra corresponding to the fern pattern. The efficacy of our procedure is demonstrated by Figure 4B where the ferning region reproduces the optical image quite well.

**Figure 5:**
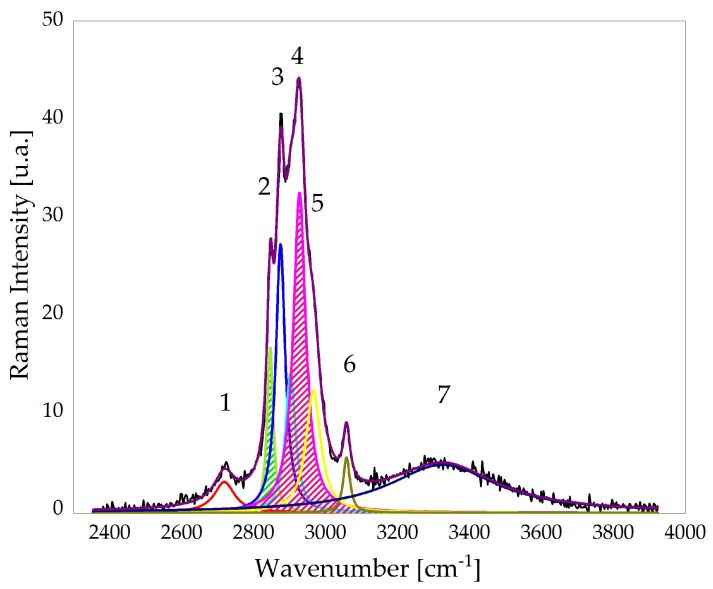
Deconvolution of the typical Raman spectrum of the fern region. The multiple peak fitting was performed in the spectral region 2300–3900 cm${}^{-1}$ by using seven Lorentzian functions. Green and pink curves correspond to the Raman peaks associated to lipids and proteins, respectively, used to evaluate the ratio *R* defined in the text.

The Raman spectra selected according to the procedure just described were used to evaluate the relative intensity of Raman bands associated with proteins and lipids. According to the literature, the ratio $R=I_{2930}/I_{2845}$ can be used to monitor the alteration of lipids and protein content in bio-samples [28]. Therefore, we proceeded by analysing this ratio for samples acquired on different days. For this purpose, the spectral region 2800–3000 cm${}^{-1}$ was fitted with seven Lorentzian curves. This procedure was repeated for all the spectra acquired in maps. A typical outcome is shown in Figure 5. In this figure, green and pink Lorenzian peaks correspond to the Raman bands associated to lipids and proteins, centred at 2845 and 2930 cm${}^{-1}$, respectively, and used to evaluate the ratio *R*. In particular, *R* was evaluated through the ratio of the areas underneath the two considered peaks (see dashed areas).

**Figure 6:**
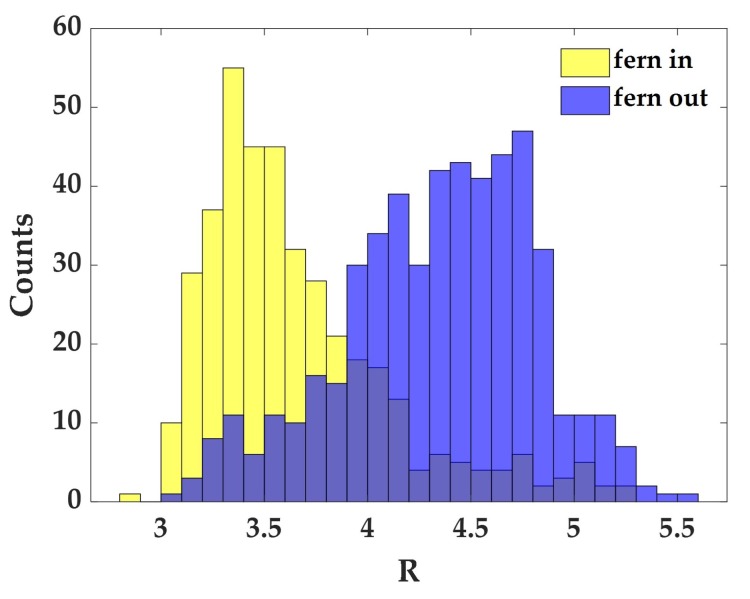
Distribution of the ratio *R* calculated for the spectra relative to yellow and blue region of the corresponding mask (Figure 4B).

In Figure 6, we report the ratio *R* obtained in a single scan from spectra corresponding to fern points (yellow bars) and to points not occupied by ferns (blue bars). As can be seen, the two distributions, although partially overlapped, present a slight different average value as a consequence of the spatial segregation of the biochemical components in the pattern. Notably, the average value of these two distributions derived from different Raman maps in the same sample are always consistent with each other, an outcome which confirms the effectiveness of our procedure to obtain reliable information on the sample biochemical content.

**Figure 7:**
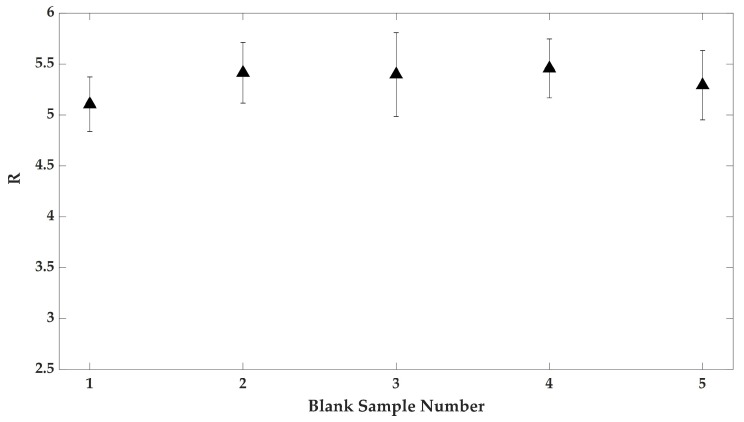
Raman intensities ratio $I_{2930}/I_{2845}$ calculated in five blank samples collected from the volunteer not wearing CLs.

Therefore, having assessed our experimental procedure, we applied it to some preliminary analyses. In particular, we collected tear fluid samples in five different experimental sessions, spanning two weeks (approximately one sample collection every three days). This analysis was performed in order to exclude any influence on tear fluid composition by external factors (diet of volunteer, environment, etc.). Figure 7 shows the obtained *R* values in these assays. From these data, it is possible to note that *R* is reasonably constant (within error bars) in all the measurements.

**Figure 8:**
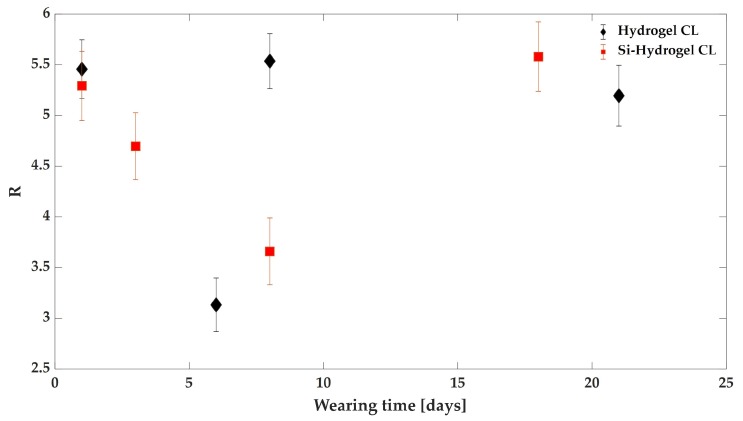
Raman intensities ratio $I_{2930}/I_{2845}$ reported as function of the wearing time for both the CLs.

After this preliminary check, we finally proceeded by analysing the effect of CL use on the tear fluid. Figure 8 reports the R values obtained for sample collected at different wearing times of the two types of CLs. According to the literature [10], some modifications in tear fluid seem to become discernible after one week of CL use, for this reason, we decided to collect samples approximately once a week. The first point in Figure 8 (day 1) refers to the blank sample, i.e., before contact lens use. The error bar refers to the mean standard deviation obtained from Raman scans of different regions of the same sample. Interestingly, the data corresponding to the Hydrogel CLs (black squares) reveal that the composition of the tear fluid is significantly affected at the beginning of the wearing period, this showing the relative content of proteins reduced with respect to lipids. It is reasonable to assume that this is due to proteins depauperation of the tear fluid as a consequence of the proteins deposition on the CLs. After this initial phase, it is possible to observe a substantial recovery of the initial tear fluid composition. This outcome could be due to a sort of partial saturation of the CLs surface, so that it is less prone to collect proteins from the tear fluid. However, in principle, the recovery of the R values might also be due to an increase in lipid deposition on the CLs [7] (which could reduce lipids in the tear fluid) but could also be caused by a physiological response from the eye to the presence of the CLs [5]. Interestingly, independently from the mechanism ruling the modification of tear fluid composition, the original mixture of the biochemical components substantially recovered after 15 days of CLs use. The same analyses were performed for the Si-Hydrogel-based CLs. As it is possible to see in Figure 8, the trend of the *R* ratio is roughly conserved, with an initial decrease of this parameter and a substantial recovery after 21 days of CLs use. Clearly, our results for the two CLs types are not representative of the generic CLs user. In fact, this is strictly dependent on the volunteer, and, in principle, on factors related to the volunteer’s health during use. This is the main reason that we decided to perform this study on tear fluid collected from only one volunteer. A much wider statistical base would be necessary to investigate, e.g., the effect of a specific CLs type (once the capability of RS in terms of revealing tear fluid modification induced by CLs has been assessed). However, this last issue was outside of the goals of our investigation.

## 4. Conclusions

The present study is a Raman-based investigation of the tear fluid of a CLs wearer. The study was performed for two types of CLs worn by the same donor in order to eliminate possible interference due to the individual response to CLs. Our results show a decrease in the relative content of proteins with respect to lipids in the first days of CLs use, followed by a substantial recovery after ≈20 days. This paper demonstrates that Raman spectroscopy is a useful tool to monitor the biochemical change induced by CLs use. To this end, we think that the results we are presenting can be considered a preliminary investigation promoting the development of a real sensor devoted to the analysis of the influence of CLs use on tear fluid composition.

## Figures and Tables

**Table 1:** Main characteristics of contact lenses (CLs).

**Proprietary Name**	LAC Monthly Wet	AirHydra
**United States Adopted Name (USAN)**	Wetafilcon	Genifilcon A
**Classification**	Hydrogel	Silicon Hydrogel
**Water Content [%]**	55	45
**Dk/t**	19.5	70.0

**Table 2:** Raman assignment of the spectral intensities in the typical tear spectrum.

Wavenumber (cm${}^{-1}$)	Assignment	Wavenumber (cm${}^{-1}$)	Assignment
1000	Phe	1605	Phe
1064	Skeletal ν(CC)(l)	1665	Amide I
1206	Aromatic Amino Acids	2845	$\nu_{s}$(CH${}_{2}$)
1242	Amide III	2875	$\nu_{as}$(CH${}_{2}$)
1296	τ(CH${}_{2}$)	2930	$\nu_{s}$(CH${}_{3}$)
1301	CH${}_{2}$ deformation (l)	2960	$\nu_{as}$(CH${}_{3}$)
1440	δ(CH${}_{2}$)(p,l),δ(CH${}_{3}$)(p)	3057	aromatic ν(CH)
1553	Trp	3300	ν(OH)

Note: Phe: phenylalanine; Trp: tryptophane; p: proteins; l: lipids; ν: stretching; δ: bending; τ: twisting.

## Abbreviations

The following abbreviations are used in this manuscript:

CLs	Contact Lenses
RS	Raman Spectroscopy
DCDRS	Drop Coating Deposition Raman Spectroscopy
H-CLs	Hydrogel Contact Lens
SH-CLs	Silicon Hydrogel Contact Lens
